# Reminders of behavioral disinhibition increase public conformity in the Asch paradigm and behavioral affiliation with ingroup members

**DOI:** 10.3389/fpsyg.2015.00837

**Published:** 2015-06-29

**Authors:** Kees van den Bos, E. A. Lind, Jeroen Bommelé, Sebastian D. J. VandeVondele

**Affiliations:** ^1^Department of Social and Organizational Psychology, Utrecht University, Utrecht, Netherlands; ^2^School of Law, Utrecht University, Utrecht, Netherlands; ^3^Fuqua School of Business, Duke University, Durham, NC, USA

**Keywords:** threats, social interaction, behavioral disinhibition, affiliation, conformity, behavior, sense-making

## Abstract

This paper argues that being in the Asch situation, where there is a felt need to conform to others’ faulty behaviors, poses a social threat to people. Furthermore, participating in a psychology experiment in which you will have to interact with other participants might trigger sense-making processes. The paper proposes that these assumed threats or sense-making processes are likely to activate the behavioral inhibition system, making people respond in more inhibited ways than they normally would be inclined to do. As a result, people’s tendency to affiliate behaviorally with persons who are similar to them can be inhibited. The implication is that lowering behavioral inhibition (by experimentally reminding people about having acted without behavioral inhibitions) should lead to more public conformity in the Asch situation and stronger behavioral affiliation with ingroup members than not being reminded about behavioral disinhibition. Findings of four experiments support this line of reasoning. These findings are discussed in terms of behavioral inhibition and behavioral affiliation. Alternative accounts of the data that focus on social belongingness threats and optimal distinctiveness are also considered.

## Introduction

In the present paper we examine the dynamics of how people respond to potentially threatening social interactions. In particular, we focus on reactions in the Asch (1951, 1955, 1956) situation, in which there is pressure to conform to the faulty answers given by other research participants. We also examine responses in psychology experiments in which people expect to interact with other participants. We argue that these kinds of social interaction situations may contain threatening or sense-making aspects for research participants and that the social interaction threats or sense-making processes may activate the behavioral inhibition system (BIS: Carver and White, 1994). We test behavioral implications of this line of reasoning by assessing how lowering behavioral inhibition by means of experimental manipulation may affect public conformity and behavioral affiliation with the other participants in the experiments. We ground our predictions by building on work on behavioral affiliation and associated literatures and aim to integrate these insights with our recently developed perspective on behavioral inhibition and disinhibition (Van den Bos and Lind, 2013).

### Behavioral Affiliation

Humans are social animals, creatures that are highly interactive with, and responsive to, other members of their species (Aronson, 1972). One of the core needs of humans is the need for affiliation. “Affiliation is the act of associating or interacting with one or more other people” (Leary, 2010, p. 865). Thus, people who want to affiliate seek to be in the company of others and want to interact with these other persons.

Theorists have suggested that being with or interacting with other people is a fundamental social behavior (e.g., Murray, 1938), especially when this involves individuals who are similar to us (Schachter, 1959). Therefore, peers or others who are similar to us serve a special function in fulfilling the need for affiliation and, more generally, people’s pursuit of interpersonal connection (Sherif and Sherif, 1964; Schwarz, 1973; Wolf, 2008; Sundar et al., 2009; see also Erikson, 1968).

Peers are those individuals who share a similar or equal status, are usually of roughly the same age, and often have similar interests and backgrounds, bonded by the premise of sameness (Wolf, 2008). These individuals have a significant influence on the behaviors of people, especially when people are high in need for affiliation (Leary, 2010). For example, members inside peer groups learn to develop relationships with others in a social system. Furthermore, peers, and in particular ingroup peers, constitute important social referents for conveying customs and social norms (Clausen, 1968). Moreover, research suggests that peers exert stronger influence on what people do than do other important figures such as authorities. For example, a correlational study by Schwarz (1973) suggested that peers have stronger effects on inmates’ behaviors than do prison authorities. An experiment on consumer attitudes by Sundar et al. (2009) showed that peer cues are generally more persuasive than are cues received from authorities or experts with high source credibility. Similarly, Harris (1995) concluded in her review article that peers may be more important for the socialization of children than are parents and other authorities.

With the phrase “The Social Animal,” Aronson (1972) highlighted that we humans have a social nature and that we have a strong tendency to affiliate with others around us, including (and probably especially) with those who are similar to us (Schachter, 1959; see also Murray, 1938; Sherif and Sherif, 1964; Clausen, 1968; Erikson, 1968; McClelland, 1987; Baumeister and Leary, 1995; Wolf, 2008). As a result of this social quality of humans, people’s behaviors tend to be influenced heavily by their social surroundings. In other words, a great many human behaviors are, at their core, socially oriented behaviors.

This does not mean, though, that socially oriented behaviors are always good or benign. In fact, the notion that our susceptibility to social influence can yield both positive and negative effects on what we do forms a central part of classic and modern social psychology. The potentially deleterious effect of peer pressure is well-known and includes instances where an individual feels directly or indirectly pressured into conforming with the group to make their behavior match that of their peers, even when conformity has a less than positive impact (Erikson, 1968; Sherif and Sherif, 1968). And in his APA-medal winning book Aronson (1972) clearly pointed out that social behaviors include not only prosocial behaviors (such as helping in bystander situations or fighting injustice), but also less benevolent behaviors (such as behaviors pertaining to prejudice, aggression, and conformity with wrong answers given in the Asch paradigm). The current paper aims to address both aspects of social influence.

A key feature of the argument we present here is that humans have a natural tendency to want to affiliate with people similar to them and that when confronted with threats people often want even more to affiliate with similar others. However, to fully understand people’s responses to threats, we also need to consider those threats that result from those situations in which we have the feeling we do not belong in the group or in which our feelings of optimal distinctiveness (e.g., Brewer, 1991) are threatened. There is a truly substantial amount of research on these kinds of threats in social psychology. For example, people feel threatened if they are socially excluded (e.g., Eisenberger et al., 2003) and react with all sorts of defenses to social exclusion (see, e.g., Baumeister and Tice, 1990; Twenge et al., 2001; Abrams, 2005; DeWall and Baumeister, 2006; Bernstein et al., 2008; Lakin et al., 2008; Molden et al., 2009; Aydin et al., 2010; Gunther Moor et al., 2011; Riva et al., 2012; Schaafsma and Williams, 2012). Furthermore, Simon et al. (1997) have shown that a mortality salience threat can lead people to want to be similar or dissimilar to others depending on whether their optimal distinctiveness to others had been threatened (i.e., whether their uniqueness or their similarity to others had been threatened). Thus, many issues need to be considered to provide a complete picture of the need for affiliation and people’s responses to threat.

Obviously, the current paper cannot address all aspects of responses to social threats. Therefore, based on notions such as peer group affiliation (Sherif and Sherif, 1964), affiliation motivation (McClelland, 1987), and the social animal (Aronson, 1972), the present paper notes that a core issue in classic and contemporary social psychology is trying to understand when people want to be involved with their fellow companions in their surroundings, and what different forms of behaviors people may engage in when they want to be involved with these peers. The current paper focuses on these issues by examining the effects of reminders of behavioral disinhibition on conforming and affiliating with peers.

### Responding to Threats in Social Interactions

One reason why people affiliate with others is to obtain relief from stressful or fearful situations (Hill, 1987). Thus, behavioral affiliation is a response often seen when people are responding to social threats. Furthermore, Schachter (1959) proposed that people who are uncertain about the nature of a situation and how they should react desire to affiliate with other people to find out (see also Leary, 2010). Therefore, following the literature on behavioral affiliation, we focus in the present paper on how people respond to threatening situations and situations in which they are at least somewhat uncertain as to how they should behave exactly.

We examine these issues by relying on recent insights that suggest that in many situations people can be surprised by what is happening and do not know how to respond to the situation at hand (see, e.g., Van den Bos et al., 2011b; Van den Bos, 2013; Van den Bos and Lind, 2013). We argue here that in these confusing situations the BIS will be activated such that people will inhibit behavioral action because they are seeking first to find out what is going on and what behavior is appropriate in the situation at hand. After people have made sense of the situation the inhibition system is deactivated and the behavioral activation system is turned on so that people can perform the behavior that they think is appropriate in the current situation (Van den Bos, 2013). We ask what implications this line of reasoning can have for our understanding of how people affiliate with and conform to peers or fellow research participants.

Asch (1951, 1955, 1956) showed that participants in his classic conformity experiments were trying to sort out what was going on in the experiments and why their fellow research participants suddenly gave wrong answers to objectively simple questions. Given that people devalue, dislike, and reject those who do not conform to their judgments, decisions, and behaviors (Schachter, 1951), people understandably conform to others’ views (Cialdini et al., 1991; Leary, 2010). Furthermore, consider the situation of a participant entering the psychology laboratory in which they are told that they will have to interact with other participants. It is a well-known fact that people who do this are trying to sort out what is going on in the experiment in which they are participating and to make sense of the situation in which they now find themselves, in particular when they will have to interact with an experimenter and other participants in the experiment. As a result of these sense-making processes, research participants are susceptible to how they are evaluated by important persons present in the lab setting. These important others may include the experimenter (Cottrell et al., 1968; Cottrell, 1972) but may also include the participants’ peers (Innes and Young, 1975).

We assume that the social threats encountered in the Asch situation as well as the more general sense-making processes triggered in psychology experiments in which you will have to participate with other participants inhibits your reactions. Our assumption is based in part on the insight that evaluation apprehension involves anxiety (Christensen, 1982) and fear of negative evaluation (Rosenberg, 1980), which are concepts that are related to the activation of the BIS (Gray, 1987; Gray and McNaughton, 2000). Asch (1951, 1955, 1956) showed that participants in his conformity experiments were trying to sort out what was going on in the experiments and why their fellow research participants suddenly gave wrong answers to objectively simple questions. Thus, in addition to anxiety and fear of negative evaluation, more general processes of sense-making play a role in how research participants act in (at least some) psychology experiments, particularly those experiments in which participants interact with others.

Here we acknowledge that there are different perspectives on the functioning of the BIS in the research literature (see, e.g., Latané and Nida, 1981; Gray, 1987; Monteith, 1993; Carver and White, 1994; Gable et al., 2000; Gray and McNaughton, 2000; Nigg, 2000; Sawyer and Behnke, 2002; Carver, 2005; Knyazev et al., 2006; Amodio et al., 2008). This noted, there is good evidence that the BIS is activated when people are faced with anxiety-triggering stimuli (e.g., Carver and White, 1994; Gray and McNaughton, 2000) or, more generally, with social situations that instigate processes of sense-making (e.g., Gable et al., 2000; Van den Bos, 2013). For example, Carver and White (1994) argue that the BIS regulates people’s responses to anxiety-related cues and inhibits behavior that can lead to negative or painful consequences. Furthermore, the BIS has also been used to explain self-regulation and inhibition of prejudiced responses (Monteith, 1993). Moreover, the BIS has also been linked to more general sense-making processes in social contexts, such as how people deal with novelty in their environments (Gable et al., 2000) or how they interpret and react to puzzling situations (Van den Bos et al., 2011b; Van den Bos, 2013).

Importantly, as explained in detail in Van den Bos and Lind (2013), our ideas about inhibition and disinhibition focus on behavioral (dis)inhibition in public contexts. We note that an important notion in social psychology is the idea that in public settings the presence of others can constrain people from following their personal inclinations. Thus, we argue that issues of *public* and *behavioral* inhibition are important elements in the psychology of inhibition and sense-making. *Public* because the inhibition of primary importance is often instigated by thoughts of what others will think of our actions in non-private and fundamentally social contexts, and *behavioral* because the main consequence of interest in our line of work will be the effects of inhibition on the behaviors that people subsequently show. In the current research we examine how this analysis may contribute to insights about when people affiliate with and conform to their fellow research participants.

### The Current Research

In the present paper we aim to combine the insights on conformity (Asch, 1951, 1955), behavioral affiliation (Schachter, 1959; Leary, 2010), and associated literatures (Murray, 1938; Sherif and Sherif, 1964; Clausen, 1968; Erikson, 1968; Aronson, 1972; McClelland, 1987; Wolf, 2008) with the idea that people try to make sense of their surroundings, including psychology experiments in which they are taking part with other participants (Cottrell et al., 1968; Rosenberg, 1980; Christensen, 1982; Geen, 1983, 1985; Van den Bos, 2013). Specifically, we attempt to integrate these insights with recent work that suggests that people in many social situations are inhibited from showing important social behaviors (Van den Bos, 2013). That is, we argue that if participants in psychology experiments in which they are expecting to interact with others indeed are inhibited from showing their social behaviors, as has been suggested in recent papers (Van den Bos et al., 2009, 2011b; Van den Bos, 2013), and if young people such as university students are indeed oriented toward their peers, as important scholars have argued (Schwarz, 1973; Harris, 1995; Sundar et al., 2009), then it should be the case that lowering behavioral inhibition will lead people to show increased affiliation with peers or others who are close or similar to them. Our previous research shows that behavioral inhibition can be lowered by reminding people of times in the past when they acted without inhibitions (Van den Bos et al., 2009, 2011b). Thus, reminding people of past disinhibited behaviors should lead them to affiliate more (not less) with their peers.

In four studies we examine the implications of this hypothesis on the actual behavior of research participants. To connect our research directly to the influence of social threats we focus in Studies 1 and 2 on people’s behavior in the Asch (1951, 1955, 1956) paradigm. That is, in Studies 1 and 2 we argue that if reminders of behavioral disinhibition indeed lead people to affiliate more with their peers, they should be willing to conform more with what their peers do. Indeed, we reveal in Studies 1 and 2 that reminding people of having acted without inhibitions leads them to conform more (not less) with the wrong answers given by fellow research participants in the Asch paradigm.

We then use Studies 3 and 4 to generalize the effects of disinhibition to other measures of peer affiliation. In particular, in Studies 3 and 4 we note that increased affiliation with peers should be shown in university students wanting to sit closer to a fellow student from their university (cf. Macrae et al., 1994; Van den Bos et al., 2007). Indeed, in Study 3 we reveal that reminding university students of having acted without inhibitions leads them to sit closer to a fellow research participant, and not closer to the experimenter. Furthermore, in Study 4 we show that reminders of behavioral disinhibition lead students to sit closer to a student from their own university, and not closer to a student from a rival other university. Thus, taken together, our four studies reveal that reminders of behavioral disinhibition increase public conformity in the Asch paradigm and behavioral affiliation with ingroup members.

In all four studies we use a behavioral disinhibition manipulation that we developed and validated in earlier research (see Van den Bos et al., 2009, 2011b). Our manipulation asks participants in the disinhibition condition to answer three simple open-ended questions that remind them about their thoughts and feelings about having behaved without inhibitions. In the control condition participants answer similar questions that do not remind participants about disinhibited behaviors.

Van den Bos et al. (2009) showed that this way of reminding (vs. not reminding) participants of having acted without behavioral inhibitions successfully lowers behavioral inhibition as assessed by a state version of the popular and well-validated measure of BIS sensitivity by Carver and White (1994). Specifically, after completing the three disinhibition questions or the three control questions, participants completed the following seven state BIS items. Following Carver and White (1994) these items asked participants to indicate to what extent they agreed or disagreed with the following statements: “At this moment, I worry about making mistakes”; “At this moment, criticism or scolding would hurt me quite a bit”; “At this moment, I would feel pretty worried or upset when I think or know somebody is angry at me”; “At this moment, I do not experience fear or nervousness, even when something bad is about to happen to me” (reverse coded); “At this moment, I would get pretty worked up when I would know that something unpleasant is going to happen”; “At this moment, I would feel worried when I would think I have done poorly at something”; “At this moment, I have very few fears compared to my friends” (reverse coded). All items were answered on 7-point scales (1 = *strongly disagree*, 7 = *strongly agree*). Reliability of the resulting state scale BIS scale was good (Cronbach’s alpha = 0.76). Results reported in Van den Bos et al. (2009) showed that the disinhibition manipulation successfully lowered behavioral inhibition such that participants in the disinhibition condition experienced significantly lower levels of state behavioral inhibition than participants in the no-disinhibition condition.

Furthermore, the disinhibition manipulation yields effects comparable to differences on Carver and White’s (1994) measure of trait BIS (see Van den Bos et al., 2011a,b). In addition, the manipulation does not trigger behavioral activation [no effects were found on state versions of Carver and White, 1994, behavioral activation scales (BAS)] nor does it influence positive or negative affective states [no effects were found on the positive and negative subsets of the Positive and Negative Affect Schedule (PANAS) by Watson et al., 1988; see Van den Bos et al., 2009, 2011b]. Moreover, the manipulation does not affect self-monitoring nor experienced accountability or self-awareness (Van den Bos et al., 2011b). Participants in studies using this manipulation typically indicate no suspicion of the procedures employed during the disinhibition manipulation nor do they suspect a direct relationship between the manipulation and their subsequent reactions in other parts of the experiments (Van den Bos et al., 2009, 2011b). Furthermore, the effects of the disinhibition manipulation can be found both among students in the psychology laboratory and in non-student samples outside the psychology laboratory (Van den Bos et al., 2009, 2011b). And in all the studies that have used this manipulation, gender did not interact with the effects of the disinhibition manipulation. Gender also does not affect the findings we will report here.

Thus, the reminders of behavioral disinhibition that we use in our studies have been pretested extensively. These earlier tests show that this is a manipulation that is conceptually related to the BIS as defined by Carver and White (1994; see also Van den Bos, 2013), significantly lowers state behavioral inhibition (Van den Bos et al., 2009), yields comparable effects as associated individual difference variables (Van den Bos et al., 2011a,b), and does so without affecting alternative concepts such as behavioral activation, affective states, self-monitoring, or accountability (Van den Bos et al., 2009, 2011b). What effects do these reminders of behavioral disinhibition have on conformity and affiliation with peers?

## Study 1

In Studies 1 and 2 we examine whether reminding people of having acted without inhibitions lead them to conform more in public with the wrong answers given by other participants in the Asch (1951, 1955, 1956) paradigm. In Study 1, participants completed reminders of disinhibition or no disinhibition, after which they were asked to participate in a human perception task. In this task, participants were asked to indicate publicly which of three lines was equal in length to stimulus lines. In the condition in which confederates were present, four other supposed participants gave wrong answers in 10 critical trials. We assessed how many wrong answers the actual participants gave during the critical trials. In Study 1, we compared these responses with answers given in a condition where no confederates were present.

### Method

#### Participants and Design

Eighty-six students (31 men and 55 women) at Utrecht University participated in the study and were randomly assigned to one of the cells of the 2 (confederates: present vs. absent) × 2 (behavioral disinhibition: disinhibition vs. no disinhibition) factorial design.^1^^,^^2^ Participants received 3 Euros for their participation in the study.

#### Procedure

The experiment was presented to the participants as consisting of two unrelated parts. In the first part, the disinhibition manipulation took place. This manipulation used the same procedures developed and extensively pretested in earlier research (for details, see Van den Bos et al., 2009, 2011a,b). Specifically, participants were asked to complete a short questionnaire of three open-ended questions. Participants in the disinhibition condition were instructed as follows:

The purpose of this questionnaire is to assess how people react to being disinhibited, that is, how people behave when they do not care about what others think of their reactions and what feelings they then experience. To this end, please complete the following three questions: Please briefly describe a situation out of your own life in which you acted without inhibitions. Please briefly describe how you behaved in the situation in which you acted without inhibitions. Please briefly describe the emotions that you experienced when you acted without inhibitions.

In the no-disinhibition condition participants completed a short questionnaire of three open-ended questions pertaining to public transportation. Specifically, participants in the no-disinhibition condition received the following instruction:

The purpose of this questionnaire is to assess how people react to using public transportation, that is, how people behave when they use public transportation and what feelings they then experience. To this end, please complete the following three questions: Please briefly describe a situation out of your own life in which you used public transportation. Please briefly describe how you behaved in the situation in which you used public transportation. Please briefly describe the emotions that you experienced when you used public transportation.

After the disinhibition manipulation, participants were informed that the first part of the study had ended and that the second part now would begin. In this part, participants were asked to participate in a study on human perception. Based on the meta-review by Bond (2005), which shows that when three or more confederates are present the tendency to conform tends to be stable, participants in the condition in which confederates were present took part in this study together with four other participants (in reality confederates who were blind to conditions). In the condition in which confederates were absent there were no other participants.

Participants were presented a total of 17 sets of vertical lines, projected on a big white screen. Each set consisted of one stimulus line and three other lines (A, B, and C). To make our stimulus materials a bit different from the original Asch materials (which consisted of horizontal lines) we used vertical lines.^3^ The stimulus line was presented at the top of the screen and the three other lines beneath the stimulus line. After the presentation of each set of lines, participants were asked to indicate out loud which of the three other lines was equal in length to the stimulus line.

In the condition in which confederates were present, three confederates first gave their answers, after which the actual participant gave his or her answer, followed by the answer of the last confederate. As in the original Asch experiment, the confederates started by answering a few questions correctly but eventually began providing incorrect responses. That is, during 7 of the 17 trials (Trials 1, 2, 5, 8, 11, 14, and 17) the confederates gave the correct answers. During the 10 other trials the confederates gave a uniformly wrong answer. Our dependent variable assessed how many wrong answers (0–10) the actual participants gave during the 10 critical trials.

At the end of the experiment, participants were thoroughly debriefed. During debriefing, participants indicated no suspicion of the procedures employed nor did they suspect a direct relationship between the disinhibition manipulation and their reactions in the perception study.

### Results

**FIGURE 1:**
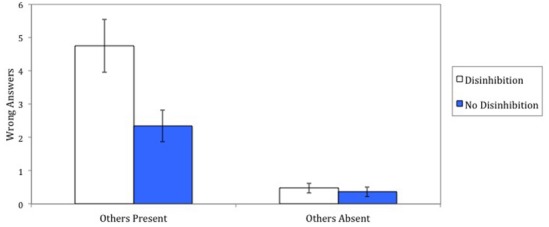
**Number of wrong answers given on critical trials as a function of other participants being present or absent and being reminded or not about disinhibited behavior (Study 1).** Error bars represent standard errors of the mean.

A 2 (confederates) × 2 (disinhibition) analysis of variance on our conformity measure (the number of wrong answers given by the participants during the critical trials) revealed a main effect of confederates being present or absent, *F*(1,82) = 62.39, *p* < 0.001, ${\text{η}}_{p}^{2}$ = 0.43, a main effect of disinhibition, *F*(1,82) = 10.11, *p* < 0.01, ${\text{η}}_{p}^{2}$ = 0.11, and a significant interaction between the confederates and disinhibition manipulations, *F*(1,82) = 8.28, *p* < 0.01, ${\text{η}}_{p}^{2}$ = 0.09. Figure 1 shows the effects together with the respective standard errors. In the condition in which confederates were present, participants gave more wrong answers when they had been reminded about disinhibited behavior (*M* = 4.75, SD = 3.19) than when they had not been reminded about disinhibited behavior (*M* = 2.35, SD = 2.16), *F*(1,84) = 6.34, *p* < 0.02, ${\text{η}}_{p}^{2}$ = 0.07. In the condition in which confederates were absent, there was no significant effect of the disinhibition manipulation, *F*(1,84) = 0.03, *p* = 0.86, ${\text{η}}_{p}^{2}$ = 0.00. Participants in this condition did not gave many wrong answers following the presence of reminders of behavioral disinhibition (*M* = 0.48, SD = 0.77) or following the absence of these reminders (*M* = 0.36, SD = 0.70).^4^

In addition, please note that when participants had not been reminded about disinhibited behavior, they gave more wrong answers following the presence as opposed to absence of confederates, *F*(1,84) = 8.81, *p* < 0.01, ${\text{η}}_{p}^{2}$ = 0.09. This replicated the original Asch finding. Furthermore, supporting our predictions, in the condition in which participants had been reminded about disinhibited behavior, the effect of confederates being present or absent was three times as large, *F*(1,84) = 37.37, *p* < 0.001, ${\text{η}}_{p}^{2}$ = 0.31, than when participants had not been reminded about disinhibited behavior.

## Study 2

Study 1 reveals that reminding people of having acted without inhibitions lead them to conform more in public with the wrong answers given by other participants in the Asch (1951, 1955, 1956) paradigm. Reminders of disinhibition do not affect participants’ line size perceptions when no other participants are present, suggesting that the effect of disinhibition reminders is not a perceptual phenomenon, but instigates increased conformity with peers.

Study 2 attempted to replicate the effect of reminders of behavioral disinhibition on conformity reactions. We did this in an experiment in which there were always three confederate participants present. After all, Study 1 showed that the effect of the disinhibition reminders was only there in the condition in which four confederates were present, and in Study 2 we wanted to see whether we could replicate the effects of the disinhibition reminders in the presence of only three confederates.

Study 2 also sought to refine our understanding of what it is in our disinhibition manipulation that causes the effect. Note that the disinhibition manipulation used in the first study reminds people of how they “react to being disinhibited, that is, how people behave when they do not care about what others think of their reactions.” This is a rather general manipulation of public behavioral disinhibition (Van den Bos et al., 2009, 2011a,b). Of course, evidence for our line of reasoning would be stronger if we could specify what this manipulation entails in somewhat more detailed terms.

In a pilot study we determined that most of our participants (Utrecht University students), when asked to indicate what they did when they acted without concerns for others present in their situation, pointed out that they voiced their own opinions in the presence of others. Voicing of opinions is an important issue to people (e.g., Hirschman, 1970; Folger, 1977; Van den Bos, 1999). In Study 2, therefore, before participants took part in the Asch paradigm we exposed them to reminders of general behavioral disinhibition, no disinhibition, or reminders of voicing their own opinions without much concerns for others present. If our reminders of general behavioral disinhibition are predominantly about disinhibition pertaining to voicing one’s own opinions, as our pilot study suggested, then the general disinhibition condition should yield about the same level of conformity as the condition in which people were reminded about disinhibited behaviors regarding voicing of their own opinions.

Building on Study 1, we again expected that the lowest levels of conformity would be shown in the absence of reminders of disinhibition. To make conforming a more attractive and easier to choose option we made the stimulus lines and the other lines more comparable to each other. Compared to Study 1 this should lead to more conformity in the no-disinhibition condition, hence providing a tougher test of our prediction that there should be more conformity in the disinhibition conditions (either general or voice disinhibition) than in the no-disinhibition condition.

### Method

#### Participants and Design

Sixty-two students (15 men and 47 women) at Utrecht University participated in the study and were randomly assigned to one of the conditions of the behavioral disinhibition manipulation (general disinhibition, voice disinhibition, no disinhibition).^5^ Participants were paid 3 Euros for their participation.

#### Procedure

As in Study 1, the disinhibition manipulation took place in the first part of the study. The no-disinhibition condition was the same as in Study 1. The instructions in the “general disinhibition” condition were the same as those used in the disinhibition conditions of Study 1. In the condition in which participants were reminded about “disinhibition regarding voice,” participants were instructed as follows:

The purpose of this questionnaire is to assess how people react to being disinhibited, that is, how people voice their own opinions in the presence of others such that they do not care about what others think of their reactions and what feelings they then experience. To this end, please complete the following three questions: Please briefly describe a situation out of your own life in which you felt no inhibitions to voice your own opinions. Please briefly describe how you behaved in the situation in which you voiced your own opinions without inhibitions. Please briefly describe the emotions that you experienced when you voiced your own opinions without inhibitions.

The second part of Study 2 was the same as in Study 1, with this time three confederate participants present in all conditions and (as in the original Asch experiment) the actual participant always being the last to answer which line resembled the stimulus line.

Participants were thoroughly debriefed at the end of the experiment. Again, participants indicated no suspicion of the procedures employed and did not suspect a direct relationship between the disinhibition manipulation and their reactions in the perception study.

### Results

**FIGURE 2:**
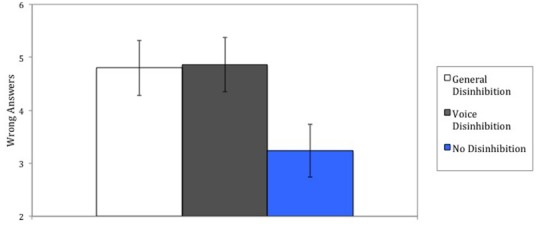
**Number of wrong answers given on critical trials as a function of being reminded about general behavioral disinhibition, disinhibition regarding voicing of own opinions, or not being reminded about disinhibited behavior (Study 2).** Error bars represent standard errors of the mean.

An analysis of variance showed a significant effect of the disinhibition manipulation on our conformity measure (the number of wrong answers given by the participants during the critical trials), *F*(2,59) = 3.31, *p* < 0.05, ${\text{η}}_{p}^{2}$ = 0.10. Figure 2 shows the effect together with the respective standard errors. When participants had been reminded about general disinhibited behavior they conformed more with the wrong answers given by the confederate participants (*M* = 4.80, SD = 2.48) than when they had not been reminded about disinhibited behavior (*M* = 3.24, SD = 1.58), *F*(1,60) = 4.72, *p* < 0.04, ${\text{η}}_{p}^{2}$ = 0.07. Furthermore, when participants had been reminded about disinhibition regarding voice they also conformed more (*M* = 4.86, SD = 2.71) than when they had not been reminded about disinhibited behavior, *F*(1,60) = 5.14, *p* < 0.03, ${\text{η}}_{p}^{2}$ = 0.08. Conformity did not differ between the general disinhibition and voice disinhibition conditions, *F*(1,60) = 0.00, *p* > 0.91, ${\text{η}}_{p}^{2}$ = 0.00.

## Study 3

Studies 1 and 2 focused on the dynamics of how people respond to threats in social interactions, in particular Asch experiments in which there is pressure to publicly conform with faulty answers of fellow participants. Study 1 demonstrated that reminding people about having acted without inhibitions lead them to conform more with the faulty answers their fellow research participants give. Study 2 replicated this effect and in addition demonstrates that similar levels of enhanced conformity are found following reminders of general behavioral disinhibition and following reminders of having voiced one’s own opinions without inhibitions.

Our line of reasoning suggested that in social threatening situations people want to behaviorally affiliate with those who are similar to them (such as their fellow research participants) but can be inhibited in showing their behavioral affiliation tendencies. After all, both behavioral affiliation (e.g., Hill, 1987) and behavioral inhibition (e.g., Gray, 1987; Gray and McNaughton, 2000) are responses that people frequently show when responding to social threats. Studies 3 and 4 aim to generalize the effects of disinhibition reminders to direct measures of peer affiliation. In particular, the student participants in Studies 3 and 4 are told that they will take part in psychology experiments with other students and we assess behavioral affiliation with peers by measuring how close our participants will sit to fellow students (Macrae et al., 1994; Van den Bos et al., 2007).

Studies 3 and 4 also extend Studies 1 and 2 by focusing on participant reactions to taking part in psychology experiments in which they merely expect to interact with other people. After all, there are many instances in which people seek to interact with others that do not involve stressful or distressing circumstances (Leary, 2010) and that involve more general sense-making processes (Van den Bos, 2013). The kind of psychology experiments on which we focus in Studies 3 and 4 may well resemble those situations that Schachter (1959) explored when noting that people who are uncertain about the nature of a situation will be motivated to affiliate with similar others to find out what to expect and how to behave in the new situation at hand.

In Study 3 we reminded our participants about times they had acted without inhibitions (disinhibition conditions) or how they act on normal days (no-disinhibition conditions). We did this using the method of earlier studies that extensively pretested this manipulation of behavioral disinhibition (Van den Bos et al., 2009, 2011a,b). After this, in a separate part of the experiment, participants were brought to a big room where they saw a small desk and a row of seven chairs. Building on and extending the method used by Macrae et al. (1994; see also Van den Bos et al., 2007), the desk at the left was where the experimenter would sit and the chair on the right was where another participant would sit. The chair in which participants sat down was our dependent variable, providing an indication of how much participants wanted to be closer to the other participant or to the experimenter.

Thus, the dependent variable was the distance, in number of chairs, between the chair with the belongings on it and the chair that the participant chose to sit on. This task measures interpersonal social distance (see Holland et al., 2004). Indeed, physical and social distances have been shown to be conceptually related (Bar-Anan et al., 2007). If our hypothesis was true that behavioral disinhibition would lead participants to want to affiliate with their peers, then we should see that reminding our student participants of disinhibited behaviors would lead them to sit closer to the other participant. In other words, we should see that reminders of behavioral disinhibition should lead to behavioral affiliation with a peer, not with an authority such as an experimenter.

Another advantage of this experimental set-up was that it allowed us to assess *behavioral* affiliation. Social psychology has always been aware that it is important to show effects of its concepts on people’s behavioral reactions (instead of only showing effects on cognitive responses, perceptions, affective reactions, or intentions), yet frequently our research does not provide behavioral data (Greenberg, 1987; Jones, 1998; Baumeister et al., 2007). Furthermore, from an applied point of view it is interesting to see whether just asking people to complete three questions that remind them of their disinhibited behaviors has behavioral consequences on where they sit down in a room.

### Method

#### Participants and Design

Sixty students (17 men and 43 women) at Utrecht University were randomly assigned to either the disinhibition or no-disinhibition conditions. Participants received 3 Euros for their participation.

#### Procedure

The experiment was presented to the participants as two separate studies. In the first study, the disinhibition manipulation was induced. The instructions in the disinhibition condition were the same as in Studies 1 and 2. Following earlier studies (Van den Bos et al., 2009, 2011a,b), participants in the no-disinhibition condition completed a short questionnaire of three open-ended questions pertaining to how they experience a normal day. Specifically, instructions were as follows:

The purpose of this questionnaire is to assess how people experience a normal day in their lives, that is, how people usually behave on a regular day and what feelings they then experience. To this end, please complete the following three questions: Please briefly describe a situation out of your own life in which you acted in a normal way like you do on a regular day. Please briefly describe how you behave when you act in a normal way like you do on a regular day. Please briefly describe the emotions that you experience when you act in a normal way on a regular day.

After the disinhibition manipulation the experimenter told participants that the first study had ended and that the second study now would begin. This second study would take place in another room across the hall. The experimenter, carrying some papers, escorted one participant at a time to this room. Upon entering the room, the participant saw a small desk and a row of seven chairs. The desk was placed at the left of the room. The experimenter put down his papers on the desk and pointed out that the desk was the place where he would sit during the second part of the study. On the right-hand chair hang a coat and below the chair there was a bag. The experimenter said: “You will participate in this study together with another student. You see the student is already there [pointing at the right-hand chair]. This student is now in the bathroom and will be back in a moment. Please seat yourself at one of the chairs and wait till the other student gets back. I will check how the other student is doing. After this, we will start the study.” The experimenter then ostensibly started walking out of the room but did not actually leave the room until the participant had sat down on one of the chairs. In this way, the experimenter could assess in which chair the participant sat (1 = *immediately next to the other participant’s chair*, 6 = *immediately next to the experimenter’s desk*) and this constituted our dependent variable.

After participants sat down, they were thoroughly debriefed. Participants indicated no suspicion of the procedures employed. Furthermore, they did not suspect a direct relationship between the disinhibition manipulation in the first study and the chair in which they sat in the second study in which they participated.

### Results

An analysis of variance revealed a significant effect of the disinhibition manipulation on our distance measure (sitting close to or distant from the other participant), *F*(1,58) = 5.97, *p* < 0.02, ${\text{η}}_{p}^{2}$ = 0.09. When participants had been reminded about disinhibited behavior they sat closer to the other participant (*M* = 2.07, SD = 0.83) than when they had not been reminded about disinhibited behavior (*M* = 2.87, SD = 1.59).

## Study 4

In accordance with our line of reasoning Study 3 reveals that reminding people of having acted without inhibitions lead them to sit closer to a fellow research participant. The reminders of behavioral disinhibition did not lead our student participants to sit closer to the experimenter. These findings suggest that disinhibition leads to behavioral affiliation with peers, not with authorities.

The aim of Study 4 was to replicate the finding that reminding people of having acted without inhibitions lead them to affiliate behaviorally with those who are close or similar to them, and not with those who are less similar to them. To this end, participants (students at Utrecht University) again first completed the reminders of disinhibition or no disinhibition, and then were asked to take a seat in a row of seven chairs. To rule out possible alternative explanations, there was no experimenter desk in Study 4 and we varied whose belongings were on the right-hand chair in the room: These belongings were said to be from another student at Utrecht University or were from a student from a rival university. If our hypothesis was true that behavioral disinhibition would lead to behavioral affiliation especially with similar people, then we should find that reminders of behavioral disinhibition will lead our participants to sit closer to the student from their own university, but not closer to the student from the rival university.

### Method

#### Participants and Design

Eighty students (25 men and 55 women) at Utrecht University were randomly assigned to one of the cells of the 2 (university affiliation of other student: same university vs. other university) × 2 (behavioral disinhibition: disinhibition vs. no disinhibition) factorial design. They received 3 Euros for their participation.

#### Procedure

The experiment was presented as two separate studies. In the first study, the disinhibition manipulation was induced in the same way as in Study 3. After this, the first study ended and the second study began. Walking to the room in which the second study would take place, the experimenter informed participants that they would participate in the second study together with another participant. The university affiliation manipulation varied whether the experimenter told our Utrecht University participants that the other participant was from Utrecht University (ingroup affiliation condition) or was from Leiden University (outgroup affiliation condition). When entering the room, participants saw a row of seven chairs. On the right-hand chair hang a coat and below the chair there was a bag. The experimenter said: “As I told you, you will participate in this study together with the other student. This student will be back in a moment. Please seat yourself at one of the chairs and wait till the other student gets back.” The chair in which participants sat down (1 = *immediately next to the other participant’s chair*, 6 = *furthest away from the other participant’s chair*) served as the dependent variable of Study 4.

After participants sat down, they were thoroughly debriefed. Participants indicated no suspicion of the procedures employed and did not suspect a direct relationship between the disinhibition manipulation and the chair on which they sat down.

### Results

**FIGURE 3:**
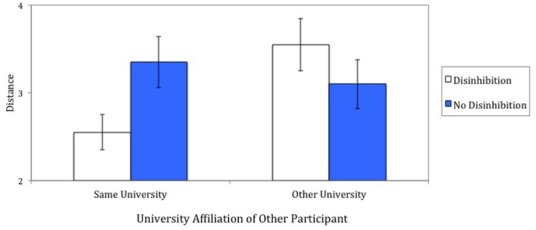
**Seating distance toward other participant as a function of university affiliation of the other participant and being reminded or not about disinhibited behavior (Study 4).** Error bars represent standard errors of the mean.

A 2 (university affiliation of other student) × 2 (disinhibition) analysis of variance on the distance measure showed only a significant interaction effect between the university affiliation and disinhibition manipulations, *F*(1,76) = 5.39, *p* < 0.03, ${\text{η}}_{p}^{2}$ = 0.07. Figure 3 illustrates the effect together with the respective standard errors. When interacting with the student from their own university, participants sat closer to the other participant when they had been reminded about disinhibited behavior (*M* = 2.55, SD = 0.89) than when they had not been reminded about disinhibited behavior (*M* = 3.35, SD = 1.31), *F*(1,76) = 4.41, *p* < 0.04, ${\text{η}}_{p}^{2}$ = 0.05. When interacting with the student from the other university, being reminded about disinhibited behavior (*M* = 3.55, SD = 1.32) or not being reminded about disinhibited behavior (*M* = 3.10, SD = 1.25) did not significantly affect where participants sat down, *F*(1,76) = 1.40, *p* > 0.24, ${\text{η}}_{p}^{2}$ = 0.02.

In addition, it is worth mentioning that in the disinhibition condition participants sat closer to the student from the same university than from the other university, *F*(1,76) = 6.89, *p* < 0.02, ${\text{η}}_{p}^{2}$ = 0.08. The effect of university affiliation was not statistically significant in the no-disinhibition condition, *F*(1,76) = 0.43, *p* > 0.51, ${\text{η}}_{p}^{2}$ = 0.01.

## General Discussion

Four experiments focused on the dynamics of how people respond to threats in social interactions. We examined this issue by means of Asch experiments in which there was pressure to publicly conform with faulty answers of fellow participants (Studies 1 and 2). We also studied behavioral affiliation by means of seating distance measures in psychology experiments in which people were expecting to interact with other participants (Studies 3 and 4). All studies demonstrate that reminding people of having acted without inhibitions leads them to show behaviors that are more oriented toward their peers, in these studies peers being fellow research participants who are similar or close to them.

In particular, Study 4 reveals that reminding people about having acted without inhibitions leads them to sit closer to fellow research participants from their own university, and not closer to those who are from a rival other university. This suggests that reminders of disinhibited behaviors lead people to affiliate behaviorally with people who are similar to them. This effect is in line with Study 3 in which we found that participants who had been reminded about behavioral disinhibition sat closer to a fellow research participant and not closer to the experimenter. Thus, Studies 3 and 4 suggest that disinhibited individuals want to affiliate with their peers, that is, that they want to be with those who are similar to them and not with those who have authority over them (Study 3) and not with those who are members from an outgroup (Study 4). Taken together, the current experiments reveal a pioneering finding that the disinhibited individual wants to affiliate behaviorally and conform his or her behaviors with those who are similar to them.

### Possible Implications

A noteworthy aspect of all our four experiments is that we obtained our effects on the actual behavior of our participants. In this way the four studies that we report contribute to pleas that social psychology should provide behavioral data (not just cognitive responses, perceptions, affective reactions, or intentions; see, e.g., Greenberg, 1987; Jones, 1998; Vohs et al., 2006; Baumeister et al., 2007). In addition, the behavioral effects we obtained on participants’ behavior are especially remarkable, we think, because we obtained them using a manipulation that consisted only of completing three questions. The findings we report here reveal that this somewhat modest manipulation yields reliable and consistent effects on participants’ actual behavior.

The findings in our first Asch experiment were obtained by contrasting reactions given in the presence of confederates who gave wrong answers with reactions given in the absence of those confederates. Importantly, the effects of our disinhibition manipulation were only found in the presence of confederates and hence only when pressure to conform to fellow research participants was high and not when this pressure was absent. Pressure to conform constitutes an important threat in social interactions and is an important reason why people affiliate with others (Hill, 1987), so this is one way in which we studied the dynamics of social threats in the current paper.

Following Schachter (1959) and others (e.g., Leary, 2010) we also examined the effects of reminders of behavioral disinhibition in situations in which we assumed that participants would be at least somewhat uncertain as to how they should behave exactly. We studied this issue in psychology experiments in which participants were expecting to interact with other participants. We note explicitly that we did not have conditions in those studies (nor in Study 2) in which the assumed social threats or uncertainties about how to behave were contrasted with conditions in which threats and/or uncertainties clearly were absent. Furthermore, the assumed threatening or uncertainty-provoking aspects of these situations were not measured in our studies. One reason for this is because it may be very difficult to reliably measure perceived threat or uncertainties (see, e.g., Van den Bos, 2009; Van den Bos and Lind, 2009). This noted, we would applaud it when future research would include more control conditions as well as sensitive measures of threat or uncertainty that would show robust evidence for the line of reasoning proposed here, for example by means of moderation or mediation analyses.

When we inspected what participants wrote down when answering the questions that asked them about their disinhibited behaviors we found that they were describing situations in which they did not feel strong public constraints on their behaviors, such as when they were attending big dance parties or other events in which they felt they could do whatever they wanted to do without others constraining their behaviors (see also Van den Bos et al., 2009, 2011a,b).

Importantly, our disinhibition manipulation does not ask participants to think about situation in which they did not follow group norms, rather it is a more general manipulation that ask them to think back about situations in which they did not care about what others were thinking of their reactions. Thus, the manipulation is not a group-related manipulation *per se*. Furthermore, when we inspected what participants wrote down when answering the disinhibition questions we did not find strong evidence that participants thought about groups and their not following group norms. Thus, we do not think the disinhibition manipulation is strongly or directly related to group behavior or group norms. We think it is better viewed of as a manipulation of interpersonal disinhibited behavior, thus behavior against other people (not necessarily groups or group members).

Previous findings have shown that our disinhibition manipulation is conceptually related to the BIS (Carver and White, 1994; Van den Bos, 2013), significantly lowers state behavioral inhibition (Van den Bos et al., 2009), yields comparable effects to those of individual differences in trait behavioral inhibition (Van den Bos et al., 2011a), and does so without engendering experimenter demands or affecting alternative concepts such as behavioral activation, affective states, self-monitoring, or accountability (Van den Bos et al., 2009, 2011a,b). Study 2 extends these findings by showing that one important component of the effect of disinhibition manipulations may have to do with people feeling free to voice their own opinions in public. The findings we present here, together with earlier research (Van den Bos et al., 2009, 2011a,b), suggest that reminders of behavioral disinhibition have conceptually meaningful and statistically significant effects on what people actually do.

In developing our ideas about behavioral disinhibition, we built our theorizing not only on work on the BIS as developed by Gray (1987; Gray and McNaughton, 2000) and Carver and White (1994), but also on the work on public inhibition as defined by Latané and Nida (1981). Latané and Nida (1981) note that in public settings the presence of others can restrain people from showing their personal inclinations. For example, in a bystander dilemma a person may want to engage in helping behavior but may be restrained from doing so because of the presence of others (bystanders) who are not helping. Similarly, we think that important elements in the psychology of inhibition and sense-making involve the issues of *public* and *behavioral* inhibition. *Public* because the inhibition of primary importance seems often to be instigated by thoughts of what others will think of our actions, and *behavioral* because the main consequence of interest in our line of work are the effects on the behaviors that people subsequently show. The studies we presented here are in line with this public and behavioral perspective on disinhibition. For example, our Studies 1 and 2 reveal that reminders of behavioral disinhibition lead to more public behavioral conformity. These findings extend insights derived from Asch’s classic experiments on public conformity and contradict common sense by revealing that it is the disinhibited participant who shows more conformity.

Earlier research has highlighted the pernicious effects of behavioral disinhibition (e.g., Newman et al., 2005) and depicted behavioral disinhibition as antisocial (Lilienfeld, 1992), psychopathological (Nigg, 2000), and a source of unwanted acts (Peters et al., 2006). Along the same lines, an important theme in moral and political philosophy has been that humans should refrain from disinhibited behavior and that it would be better for the greater good if people acted with more inhibition than they normally do (e.g., Kant, 1959). In contrast, our research program thus far has highlighted more benign effects of behavioral disinhibition. For example, we showed that following reminders of behavioral disinhibition people do not suffer from the usual bystander effects that limit helping (Van den Bos et al., 2009) and are more likely to resist advantageous but unfair outcomes (Van den Bos et al., 2011b).

Going beyond these insights, the current studies provide a more nuanced perspective on behavioral disinhibition. Yes, reminders of behavioral disinhibition can lead people to conform with faulty answers given by fellow research participants (Studies 1 and 2), but our work suggests that is it is not just the case that disinhibition provokes conformity. Rather, the link between disinhibition and conformity should be understood in light of the fact that following disinhibition reminders people want to affiliate with those who are close or similar to them (Studies 3 and 4). Thus, behavioral disinhibition is best not viewed as unequivocally bad (e.g., Kant, 1959) or antisocial (Lilienfeld, 1992), but rather should be viewed of as triggering peer-oriented responses. This can lead to benign effects, such as helping of peers in need (Van den Bos et al., 2009) or rejection of outcomes that are unfairly better than outcomes of peers (Van den Bos et al., 2011b), but also to conformity with faulty behaviors of those peers (Studies 1 and 2). Behavioral disinhibition as a trigger of increased peer affiliation yields a new, more precise, and more nuanced understanding of behavioral disinhibition than seen previously in the research literature (e.g., Lilienfeld, 1992; Nigg, 2000; Suler, 2004; Peters et al., 2006; Van den Bos et al., 2009, 2011b).

### An Alternative Account of the Data: Belongingness Threat and Optimal Distinctiveness

The empirical observation that reminders of behavioral disinhibition are related to more conformity and more group affiliation is an intriguing effect. In fact, we think that the counterintuitive quality may be part of what makes this effect so interesting. This noted, the exact psychological processes instigated by our reminders of behavioral disinhibition should be examined in more detail in future research. Although earlier evidence revealed that the disinhibition manipulation attenuates a state version of the Carver and White (1994) behavioral inhibition scale, and does not influence state versions of the Carver and White BAS and also does not have reliable effects on positive and negative affective states, self-monitoring, accountability, or self-awareness (Van den Bos et al., 2009, 2011b), more insights into these issues is needed. After all, there are different conceptualizations and associated measures of behavioral inhibition and activation out there and these conceptualizations and measures may well yield better insight into the exact psychological processes triggered by our disinhibition manipulation.

An important possibility that needs to be examined carefully in future research is whether being reminded of one’s own socially deviant behavior can constitute a threat to social belonging and optimal distinctiveness. After all, it could be argued that being reminded about not having cared about what others think of your reactions might lead participants to realize that there have been instances in which they acted too individualistically and did not pay enough attention to important social connections. Arguably, this might threaten the balance between people wanting to belong to important social groups and form meaningful social connections and their desire to be unique individuals who stand out a bit (but not too much) from other persons, other groups, and other social connections.

Optimal distinctiveness theory (e.g., Brewer, 1991) suggests that reminders of being individuated should increase affiliative needs and thus the enhance motivation to belong to social groups. There is certainly evidence for this effect, beginning with the work presented in Brewer’s (1991) initial article on that prominent model. Thus, our reminders of behavioral disinhibition might in fact have disturbed the balance of optimal distinctiveness and might have instigated belonging threats to at least some of the participants. Viewed in this way the effects reported in the present article would conceptually replicate studies demonstrating that after reminders of possible social exclusion people show affiliative tendencies, such as mimicry or norm conformity (see, e.g., Leary, 2010).

Thus, optimal distinctiveness and social belongingness threats may provide important alternative accounts of the findings we presented here. After all, an intriguing aspect of the current findings is that if the disinhibition manipulation induced people to feel free to voice their own opinions in public why they did not stick to the correct answer in the Asch paradigm? On the contrary, the manipulation seemed to have caused people to behave in such a way as to show a great deal of concern about social evaluation and a great deal of caring about what others think of their reactions. In short, optimal distinctiveness and social belonging may constitute important alternative accounts for explaining the effects of our manipulation.

We assumed that people are naturally inclined to affiliate with others but their natural inclination to affiliate with others can be inhibited. We further argued that the disinhibition manipulation allows participants to break free of this inhibited state and follow their natural inclination to affiliate with others. Importantly, our disinhibition manipulation (which involves having people think of a time in which they did not care about what others were thinking of their reactions) might cause an important affiliation threat to participants, which may partly help to explain our results.

Exploring the psychology of peer relations as well as group psychology may also be important in this regard. These concepts may share important similarities but may also differ in important ways from each other and understanding the similarities and differences between these concepts and related issues such as affiliation and belonging (Leary, 2010) may help to better understand the effects presented in the present paper.

To conclude this section, we strongly advocate for future research that would show whether and how our disinhibition manipulation is related to processes of social belonging and optimal distinctiveness. Obtaining these kinds of findings would elucidate the psychological processes underlying the behavioral effects reported here and elsewhere (see, e.g.,Van den Bos et al., 2009, 2011a,b; Van den Bos and Lind, 2013). In earlier research we observed that our disinhibition manipulation successfully lowers a state version of the Carver and White (1994) BIS scale, so we also suggest that our manipulation is conceptually and empirically related to behavioral inhibition in social contexts as we defined it here and elsewhere (see, e.g., Van den Bos and Lind, 2013). More fine-grained insight into the psychological processes discussed here would add a significant contribution to theory building and would help to identify a fascinating field of research.

### Other Possible Limitations

The concept of approach motivation may also help to interpret the findings reported here. For example, Harmon-Jones et al. (2013) conceptualize approach motivation as the urge to move toward something. Perhaps this suggests that our results suggest that people who overcome inhibition show approach-related behavior such that in Studies 1 and 2 people approach the opinion of their peers and that in Studies 3 and 4 people approach peers, in particular ingroup members. Thus, future research may want to focus on how approach-related behavior might be involved in the results presented here. Different operationalizations of approach motivation (see, e.g., Coan and Allen, 2003; Harmon-Jones et al., 2013) may be relevant here.

Another issue that should be examined is whether behavioral inhibition and activation are independent of each other. Many social psychologists have good reasons to consider the BIS and BAS as constituting independent systems (e.g., Carver and White, 1994; Gable et al., 2000; Gray and McNaughton, 2000), but current cognitive psychologists also tend to focus on the interaction between the BIS and BAS (e.g., Knyazev et al., 2006).

Of course, the findings of Studies 1 and 2 are limited to public conformity, which is not the same as private conformity (see Asch, 1951, 1955, 1956). This noted, Studies 1 and 2 suggest that following reminders of behavioral disinhibition people actively affiliate more with those who are similar to them, and it is certainly possible that this desire for greater affiliation will affect private, as well as public, conformity. Future research is needed to examine the effects of behavioral disinhibition on private conformity as well as its effects on additional other-oriented reactions, including relevant cognitions, feelings, and other internalized responses.

Although we believe that the effects of reminders of behavioral disinhibition are better studied using a chain of experiments (Spencer et al., 2005), rather than by attempting to tap intervening variables that may disturb the effects of the reminders, we do want to note explicitly that future research is needed to examine relevant moderators and mediators of the processes suggested by our findings. For example, in earlier research we found that differences in social value orientations moderate the effects of reminders of behavioral disinhibition on reactions to being overpaid (Van den Bos et al., 2011b). In contrast, though, social value orientations do not moderate the influence of behavioral disinhibition on reactions to bystander situations (Van den Bos et al., 2009), moral dilemmas (Van den Bos et al., 2011a), or the findings we presented in this paper. It appears that some processes, such as responses to bystander situations, moral dilemmas, behavioral affiliation settings, and conformity are so robust that they are not moderated by social value orientations, while other reactions, such as responses to being overpaid or to experiencing other mixed-motive situations, are susceptible to the moderating influence of social value orientations.

Do the findings we presented here imply that disinhibited people will seldom or never be influenced by authorities, but rather only by peers? Of course not. Research clearly shows that authorities can have strong influence on what people do (see, e.g., Cottrell et al., 1968; Milgram, 1974; Tyler and Lind, 1992). But our findings do suggest that the disinhibited individual is more likely to affiliate with their peers than with authorities (see, e.g., Study 3). Future research should examine under what conditions affiliation with authorities becomes more likely. Future research should also explore other antecedents of behavioral affiliation and conformity, such as physical similarity (Mackinnon et al., 2011) or being mimicked by others (Van Baaren et al., 2003).

## Conclusion

Building on and extending earlier work on behavioral inhibition (e.g., Latané and Nida, 1981; Carver and White, 1994; Gray and McNaughton, 2000) and behavioral disinhibition (e.g., Suler, 2004; Van den Bos et al., 2009, 2011a,b) the aim of this paper was to examine the dynamics of how people make sense of and respond behaviorally to threats in social interaction experiments. To this end, we delineated some important and unexplored effects of reminders of disinhibited behavior. In particular, we reasoned that reminders of behavioral disinhibition would want to affiliate with their peers more. Supporting this line of reasoning we found that reminders of disinhibition lead people to show more conformity with faulty answers given by their peers in the Asch paradigm (Studies 1 and 2). Our findings also revealed increased behavioral affiliation following reminders of behavioral disinhibition (Studies 3 and 4). These effects were obtained on actual behavior in both modern and classic experimental paradigms oriented toward the understanding of human behavior pertaining to public conformity (Asch, 1951, 1955, 1956) and behavioral affiliation (Macrae et al., 1994). Taken together, our studies portray the disinhibited individual as someone who in potentially threatening social interactions affiliates and conforms with his or her peers.

### Conflict of Interest Statement

The authors declare that the research was conducted in the absence of any commercial or financial relationships that could be construed as a potential conflict of interest.

## Footnotes

1 In all studies of this paper, gender was proportionally distributed among conditions. Furthermore, gender did not interact with the hypotheses under consideration and hence was dropped from the analyses.

2 We report all manipulations, all data exclusions, and all measures in our studies (Simmons et al., 2012), so we note that in Study 1, 14 extra participants took part in the experiment and were removed from the analyses reported: Three participants knew about the Asch experiments, one participant indicated suspicion about the experimental procedure used, eight participants had to omitted because faults in the experimental procedures were made when running these participants, and two participants from the no-disinhibition control condition were removed from the analyses because inspecting Cook’s (1977) distance measure in our main analysis (Cohen et al., 2003) revealed that they showed a distance score of more than 3.50 SDs above the mean. We further note that after assessing conformity in Studies 1 and 2, and measuring distance in Studies 3 and 4, participants completed some questionnaires. The results of these questionnaire findings are not discussed here and are available on request.

3 Debriefing indicated that participants of Studies 1 and 2 did not see a relationship between our studies and the original Asch (1951, 1955, 1956) experiments, with the exception of the participants mentioned in Footnotes 2 and 5 who were omitted from the analyses presented.

4 Controlling for heterogeneity using the Welch–Satterthwaite approach (Satterthwaite, 1946; Welch, 1947) yielded the same results: a significant interaction between the confederates and disinhibition manipulations, *F*(1,27.85) = 5.69, *p* < 0.03, indicating a significant effect of the disinhibition manipulation when confederates were present, *F*(1,25.30) = 6.62, *p* < 0.02, and a non-significant effect when confederates were absent, *F*(1,47.57) = 0.33, *p* > 0.56. Thus, heterogeneity does not affect the conclusions regarding Study 1. Furthermore, heterogeneity of variance was not an issue in the other experiments reported in this paper.

5 In Study 2, 14 extra participants took part and were removed from the analyses presented: Five participants knew about the Asch experiments, three participants had to omitted because faults in the experimental procedures were made when running these participants, five participants had difficulty understanding the questions asked to them, and one participant from the no-disinhibition control condition was removed from the analyses because inspecting Cook’s distance measure in our main analysis indicated that this participant showed a distance score of more than 2.75 SDs above the mean.
